# Superhydrophobicity and size reduction enabled *Halobates* (Insecta: Heteroptera, Gerridae) to colonize the open ocean

**DOI:** 10.1038/s41598-020-64563-7

**Published:** 2020-05-08

**Authors:** G. A. Mahadik, J. F. Hernandez-Sanchez, S. Arunachalam, A. Gallo Jr., L. Cheng, A. S. Farinha, S. T. Thoroddsen, H. Mishra, Carlos M. Duarte

**Affiliations:** 1 King Abdullah University of Science and Technology (KAUST), Biological and Environmental Science and Engineering (BESE) Division, Red Sea Research Center (RSRC), Thuwal, 23955-6900 Saudi Arabia; 2 King Abdullah University of Science and Technology (KAUST), Physical Science and Engineering (PSE), Thuwal, 23955-6900 Saudi Arabia; 3 King Abdullah University of Science and Technology (KAUST), Biological and Environmental Science and Engineering (BESE) Division, Water Desalination and Reuse Center (WDRC), Thuwal, 23955-6900 Saudi Arabia; 4 Scripps Institution of Oceanography, University of California San Diego, La Jolla, CA 92093-0202 USA

**Keywords:** Evolutionary ecology, Biosurfaces, Bioinspired materials

## Abstract

Despite the remarkable evolutionary success of insects at colonizing every conceivable terrestrial and aquatic habitat, only five *Halobates* (Heteroptera: Gerridae) species (~0.0001% of all known insect species) have succeeded at colonizing the open ocean – the largest biome on Earth. This remarkable evolutionary achievement likely required unique adaptations for them to survive and thrive in the challenging oceanic environment. For the first time, we explore the morphology and behavior of an open-ocean *Halobates germanus* and a related coastal species *H. hayanus* to understand mechanisms of these adaptations. We provide direct experimental evidence based on high-speed videos which reveal that *Halobates* exploit their specialized and self-groomed body hair to achieve extreme water repellence, which facilitates rapid skating and plastron respiration under water. Moreover, the grooming behavior and presence of cuticular wax aids in the maintenance of superhydrophobicity. Further, reductions of their body mass and size enable them to achieve impressive accelerations (~400 ms^−2^) and reaction times (~12 ms) to escape approaching predators or environmental threats and are crucial to their survival under harsh marine conditions. These findings might also inspire rational strategies for developing liquid-repellent surfaces for drag reduction, water desalination, and preventing bio-fouling.

## Introduction

The proliferation of invertebrate taxa in the ocean during the Cambrian explosion eventually led to their colonization on land, where insects first appeared ~479 million years (Myr) ago^1^ to eventually dominate various terrestrial ecosystems^2,3^. With an estimated ~5.5 million extant species^4^, insects comprise 80% of all known metazoans, making them the dominant animals on Earth^5^. Despite their successful colonization of both terrestrial and aquatic habitats, only a small number of insects (~0.5% of total species) have been found in marine environments, mostly in nearshore habitats, with only a handful of *Halobates* (~0.0001% of insect species) ultimately colonizing the open ocean, the largest biome on Earth^6–8^.

*Halobates* is a member of the family Gerridae, which evolved nearly 55 Myr ago^6,9^. Some 48 species of *Halobates* are known to occur in tropical and subtropical seas and oceans that cover more than 70% of the Earth’s surface. Most of these species are found in mangrove and near-shore habitats and two even live in freshwater, whereas five live on the surface of the open ocean. The fact that only a mere 0.0001% of insect species were able to colonize the largest habitat on Earth indicates that there must have been formidable environmental challenges for oceanic *Halobates* to overcome in order to live there.

*Halobates* most likely evolved from an estuarine or mangrove ancestor that was washed out to the sea and became adapted to survive in the open ocean^8,10^. Freshwater relatives of sea-skaters are often found on placid water bodies such as small ponds, lakes or slow-flowing streams, whereas many tropical species can be found on fast-flowing forest streams^11^. They use the surface tension of water to stay afloat while they feed on small invertebrates or terrestrial insects that are caught at the air-water interface^12,13^. The open ocean, on the contrary, is characterized by high turbulence, rough weather conditions, strong winds and frequent storms. Successful occupation of this hostile environment would have required *Halobates* to develop special adaptations to overcome these major environmental challenges. Although several of the adaptations required for *Halobates* to thrive in the ocean, such as feeding, prey capture^10,12,14^, reproduction^15,16^, dispersal^16^ osmotic regulation^14^ and protection from high UV^10,17^, have also been described in other Gerridae species, the fundamental question of how *Halobates* could survive storms without drowning, the key adaptation that enabled them to colonize the open ocean^18^, has remained largely unresolved.

Although it has been postulated more than four decades ago^18^ that the critical adaptation enabling *Halobates* to live on the open ocean could be attributed to its special microtrichia body covering, detailed studies of their biomechanical attributes and self-cleaning behaviors have not received much attention. This is the first experimental study to explore how *Halobates* were able to conquer the open ocean habitat where all other insects have failed. We hypothesize that in order for *Halobates* to avoid drowning, which is essential to their successful colonization of the open ocean, certain behavioral interactions associated with morphological traits in addition to physiological adaptations would be necessary. We carried out experimental analyses and observations on two species, the open-ocean *Halobates germanus* and the coastal *H. hayanus*. Here, we describe the critical set of nested morphological, mechanical, behavioral, and biochemical adaptations that led to *Halobates*’ successful colonization of the open ocean. In particular, we demonstrate, using a broad range of approaches, how these adaptations confer extreme water-repellence to allow their remarkable agility at the sea-air interface, thereby allowing them to cope with ocean turbulence and to avoid predation by fish from below and seabirds from above.

## Results and discussion

### Body morphology and superhydrophobicity

**Figure 1:**
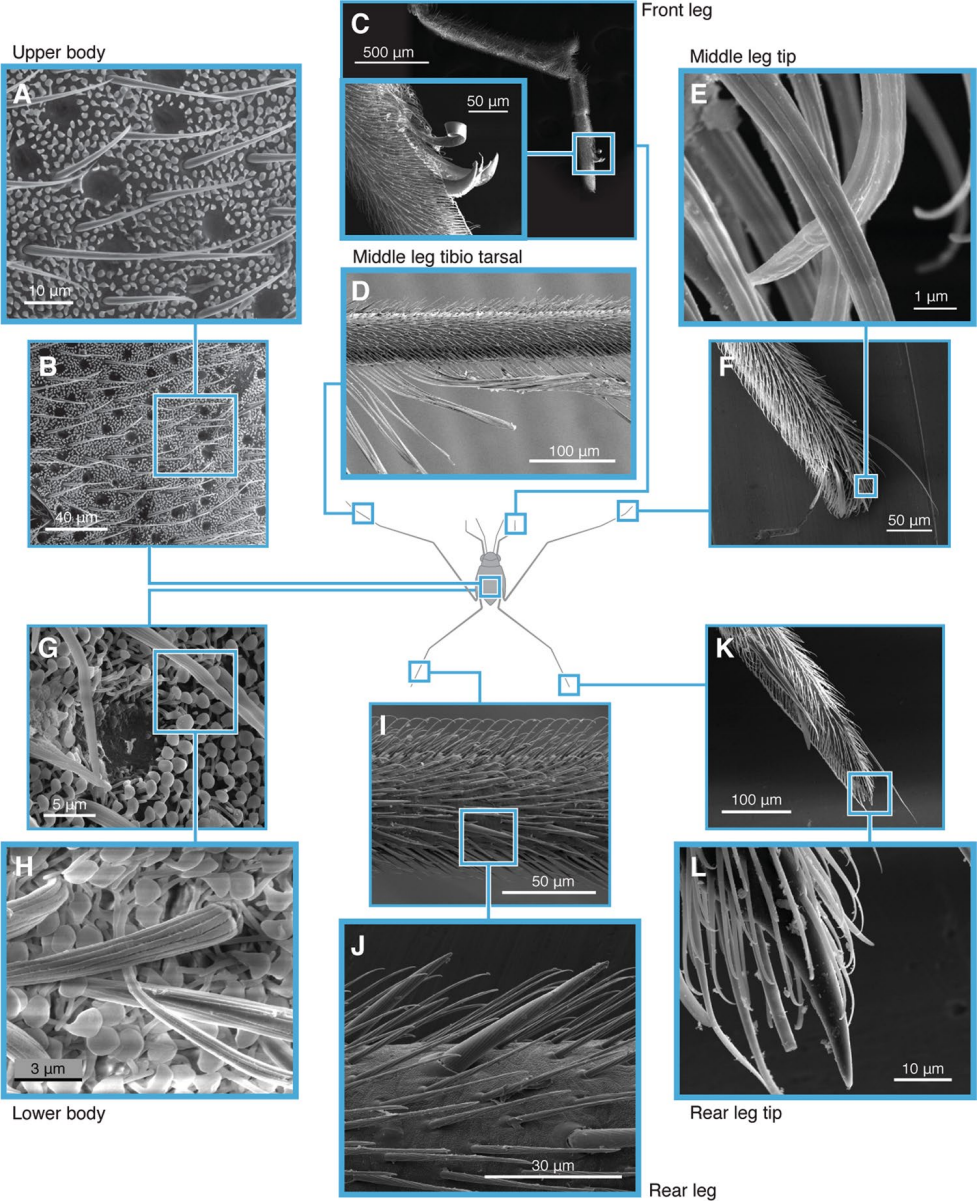
Specialized body hair of *Halobates germanus*. Scanning electron micrographs of *H. germanus*. (**A**,**B**,**G–H**) Dorsal and ventral mesothorax showing densely packed layer of tiny and fine hairs (microtrichia), interspaced with longer setae (macrotrichia). (**H**), Distinct mushroom shapes of microtrichia with broad heads that render robust wetting-resistance. Together, these hairs create an architecture that imparts multilevel protection from wetting and drowning of the insect. (**C**) Pre-tarsal claws on front leg (or ungues) used for grooming. (**D–F**,**I–L**) Middle and hind legs with an array of setae oriented at a 40° angle; hair densities vary on each segment of the leg. (**J**) Setae with longitudinal nanoscale grooves which enhance water repellency and facilitate the entrapment of air upon accidental submersion. (**I**) Ventral surface of legs that are in contact with seawater with hairs that are slightly curved at their tips, forming a continuous pane parallel to the water surface and providing greater stability. (**F**,**K)** Sensory trichobothria on legs.

The body of *Halobates* is covered by a variety of specialized hairs (Fig. 1). The very small and densely packed microtrichia (1.5 to 2 µm in length) cover much of its body (both the dorsal and ventral mesothorax, Fig. 1A,B,G,H), while the less abundant setae or macrotrichia (30 µm in length) (Fig. 1H,J) are interspersed among the microtrichia and also found on their middle and hind legs (Fig. 1E,J). Typically, the tip of a microtrichium is ~5 times wider than its shaft, rendering it like a mushroom (Fig. 1G,H), as previously shown^18^. For *Halobates*, which is restricted to oceanic life, the structure and arrangement of the characteristic mushroom-shaped microtrichia seems to be particularly crucial in the formation of a more efficient plastron as compared to the peg-like microtrichia of a related stream-dwelling *Ventidius* sp^18,19^. In comparison to pegs, mushroom-shaped microtexture have recently been shown to facilitate a significantly more robust entrapment of air upon immersion in water, regardless of the surface chemistry^20^. The fine structure of body hair layer in six families of semi-aquatic bugs, including the Gerridae to which *Halobates* belongs, has been reviewed by Andersen^13^ with detailed descriptions of their hair layer morphology. Briefly, the basic structure has two layers: a long macro-hair layer and a short micro-hair layer. The structure of the macro-hair layer, with two types of long hairs, is similar for all species except for differences in hair lengths, spacing and densities. The micro-hair layer is distributed all over the body in most species studied but differ greatly between species in size, shape and orientation (Supplementary Section S1). The density of both micro-and macro- hairs are much higher in *Halobates* than its freshwater relative *Ventidius*^13^ thus providing it with enhanced waterproofing. Functionally, the body hair of *Halobates* can therefore prevent the insect from wetting by mist or rain, which could eventually lead to accidental submersion, and facilitate a plastron that enables them to resist and emerge from accidental underwater submersion (Movie S5)^18,21,22^.

**Figure 2:**
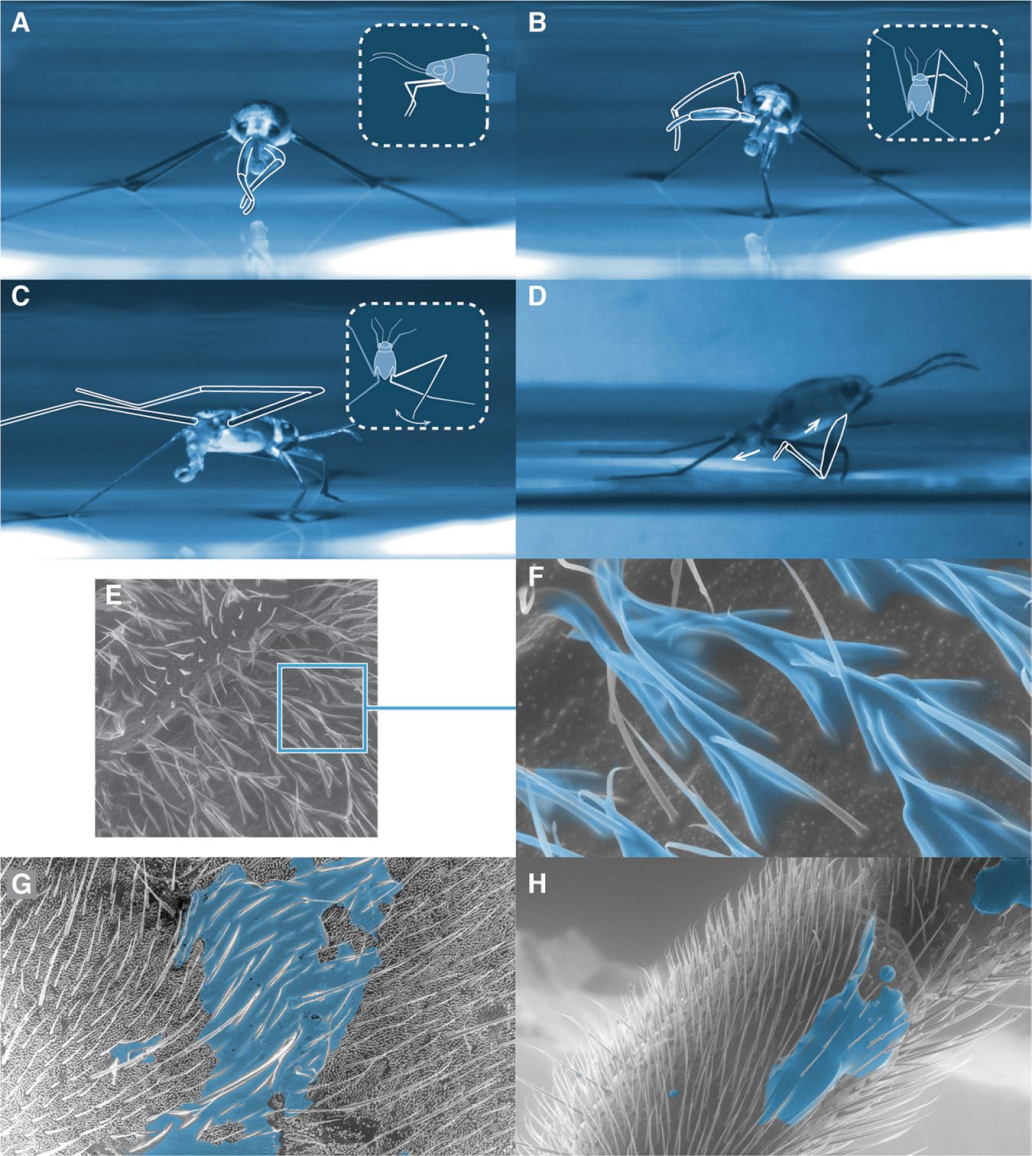
Grooming behavior of *Halobates*. (**A**–**D**) Different stages of grooming exhibited by *Halobates hayanus* with specialized activities. (**A**) Tibio-tarsal regions of the fore legs are held together and rubbed against each other; (**B**) One fore leg brushes over the posterior region of the middle leg; (**C**) Posterior regions of middle and hind legs brush against each other with the body supported by the other legs; (**D**) Fore legs brush the ventral abdominal metathoracic region alternately. (**E–H**) Transfer of waxy secretion (shown in blue) from metathoracic gland to various parts of the body.

The combination of nanoscale features on the hair (Fig. 2E,H) and waxy organics (Supplementary Table S1) confers upon *Halobates* extreme water repellence, ‘superhydrophobicity’, characterized by apparent contact angles of water droplets of size ~2 µL, *θ*_r_ > 150°, with easy roll-off on tilting of less than 20° (Supplementary Movie S1, Fig. S1). We measured apparent contact angles of water on the body and legs of the insect and found them to be *θ*_r_ ≈ 160° ± 5° (Fig. S1). To understand wetting of *Halobates*’ hairy legs by seawater under calm conditions, we utilized the Cassie-Baxter model (Supplementary Section S2) that relates the apparent contact angle, the intrinsic contact angle, and the solid-liquid and liquid-vapor interfacial areas by the relation^23^,

1 $$\cos \,{\theta }_{{\rm{r}}}={\phi }_{{\rm{LS}}}\,\cos \,{\theta }_{{\rm{o}}}\,{-\phi }_{{\rm{LV}}}$$

where *θ*_r_ is the apparent contact angle, *θ*_o_ is the intrinsic contact angle of water on a smooth and flat waxy surface (*θ*_o_ ≈ 105°)^24^, and *ϕ*_LS_ = *A*_LS_/*A*_H_ and *ϕ*_LV_ = *A*_LV_/*A*_H_ are, respectively, the ratios of real liquid-solid (*A*_LS_) and liquid-vapor (*A*_LV_) areas compared to the projected horizontal area (*A*_H_)^25,26^. Based on these values and the geometry of hairs on the legs (Fig. S2), we estimated ${\phi }_{{\rm{LS}}}\approx 5 \%$ and ${\phi }_{{\rm{LV}}}\approx 95 \%$_,_ which means that under stationary conditions *Halobates* is practically floating in air. This observation is in agreement with previous observations for *Gerris remiges* under similar conditions, where ${\phi }_{{\rm{LS}}}$ and ${\phi }_{{\rm{LV}}}$ were, respectively, found to be 4% and 96%^27,28^. The tibio-tarsal region of the middle legs, which is in contact with the water, has distinct 200 μm-long hairs (Fig. 1D), which ensures a corresponding increase in the area of contact with the water surface^10^. The tilt of these long hair may facilitate *Halobates* in detaching from the water surface and aid in skating or jumping, a feature reminiscent of the adhesion/retraction of pads in geckos^26^. Superhydrophobicity thus enables *Halobates* to skate effortlessly on water and remain dry despite being splashed by waves or under submersion, as has been shown for their freshwater relatives^28–30^ and other insects such as the brine fly *Ephydra hians* recently^31^.

### Grooming behavior

Grooming was commonly observed in *Halobates* during acclimation and imaging experiments. The insect begins grooming by resting on its middle and hind legs with its head raised and the posterior region of its abdomen, including the proctiger, remaining in contact with the water surface at an angle of 30°−40°, as it uses its forelegs to rub the abdomen (Fig. 2, Supplementary Movie S2). It comprised the following activities: (i) tibio-tarsal regions of the forelegs being held together and then rubbed against each other; (ii) one of the forelegs brushed over the posterior region of the middle leg; (iii) posterior parts of the middle or hind legs rubbing against each other, and (iv) both forelegs brushed under the abdominal and meta-thoracic regions alternately (Fig. 2 and Supplementary movie S2). When exposed to intermittent sprays of water droplets or simulated splashing waves, *Halobates* instantaneously responded by avoiding them through persistent jumps. Grooming activity increased significantly as water droplets accumulated on the body surface. As the spraying continued further, it performed a somersault, thus shedding water from its body (Supplementary movie S3). Because these grooming behaviors were carried out continuously with only short pauses between any two activities, it was not possible to identify any particular order. In the 25 video sequences analyzed, we found that activity (i) was the most prevalent, followed by (ii) and (iii), while (iv) was relatively rare. We did not notice any significant difference between the grooming behaviors of *H. germanus* and *H. hayanus*.

We speculate that grooming serves multiple purposes in (a) maintaining alignment of the setae and macrotrichia which imparts superhydrophobicity (b) facilitating removal of water droplets from the body surface that contributes to rain-proofing (c) removing contaminants from the body surface which could otherwise compromise superhydrophobicity and (d) allowing redistribution of wax from the ventral meta-thoracic gland opening to different parts of their body.

To characterize the nature of the material produced by *Halobates* to coat its body, we collected and extracted the chemical from 450 *H. hayanus* adults and analyzed it with gas chromatography (Methods). The cuticular wax of *Halobates* was found to be a complex mixture of long-chain hydrocarbons (C_9_–C_28_ alcohols, alkanes, alkenes, dienes, esters, and ketones), with molecular weights ranging from 138–410 amu (Supplementary Table S1). Several of these hydrocarbons are known to be highly hydrophobic in nature and could contribute to superhydrophobicity and self-cleaning^32^ as well as serving other biological functions such as protection against pathogens^33^ and chemical communication^34^ as observed in other insects. Curiously, even after the removal of wax by organic solvents, the dead *Halobates* still exhibited superhydrophobicity, perhaps due to their chitin-based exoskeleton.

### Dynamic agility of *Halobates*

**Figure 3:**
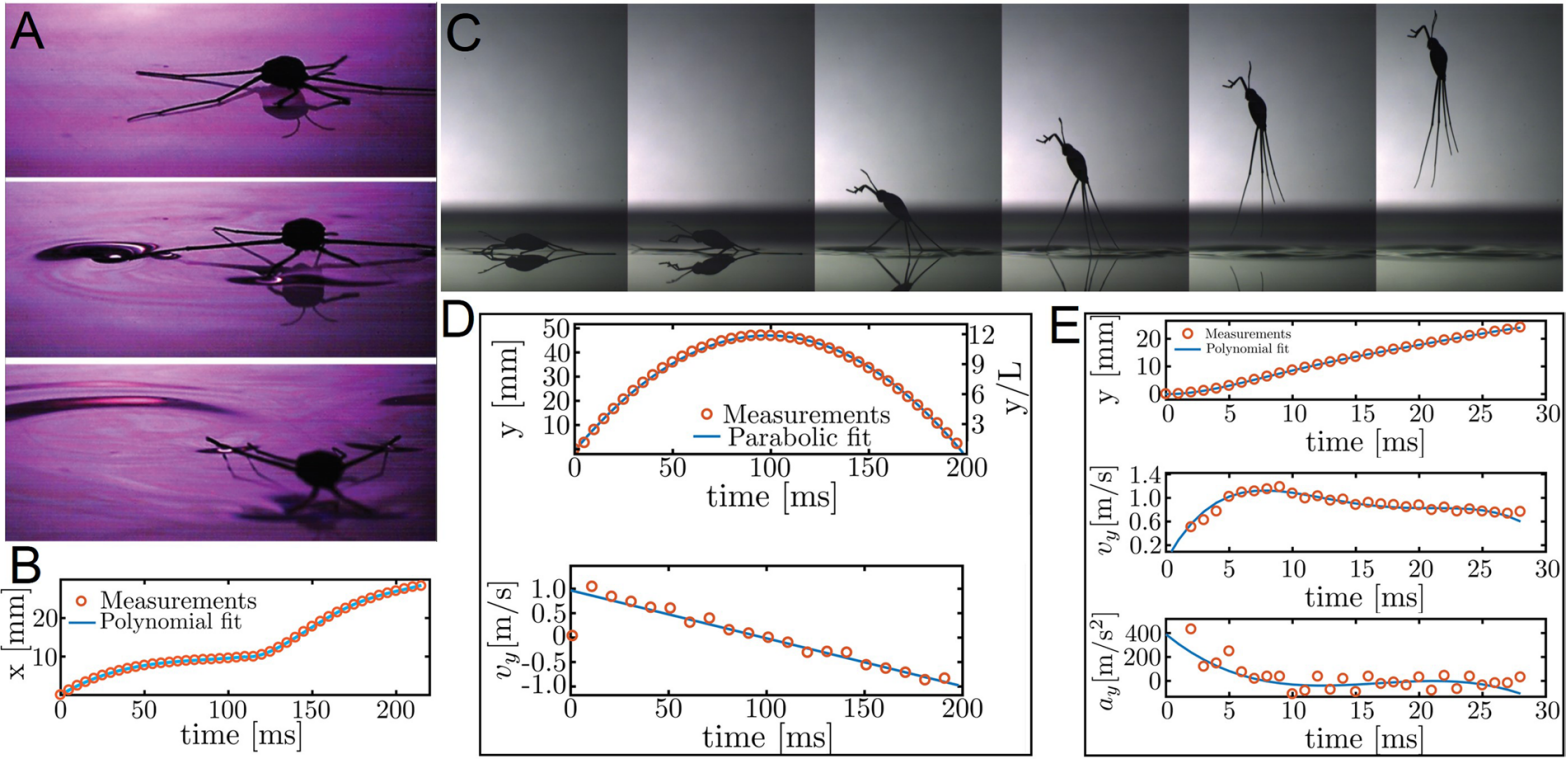
Dynamics of locomotion of *Halobates germanus*. (**A**,**B**) Insect shown skating over the water surface. In a static state, the insect’s weight is supported by its middle and hind legs by exploiting the surface tension of the water. The insect moves on the water surface by rowing its middle legs to generate momentum to propel it forward. The flow generated during this driving stroke is visible as a wave-field disturbance. (**C**) During a vertical jump, the middle and hind legs press on the water surface to create dimples, which maximize the upward propulsive force before take-off. The legs are then pulled towards the body, resulting in a vertical high-velocity jump that propels the insect several body lengths upwards in the air. (**D**,**E**) Graphs showing changes in kinetic parameters during a jump and take-off, respectively.

The vertical jumping agility of *Halobates* on water is also facilitated by the insect’s superhydrophobicity. The specialized morphology of their legs combined with the alignment of the hair fringe prevent the tip of their legs from piercing the meniscus of the water (Fig. 3), as reported previously for freshwater striders *Gerris latiabdominis*, *G. gracilicornis* and *Aquarius paludum*^35,36^.To avoid danger, such as breaking waves or predators, the insect initiates a jump by pushing its middle and hind legs against the water surface to creating dimples on the water surface (Fig. 3C, Supplementary movie S4). The curvature of the air-water interface (dimples) enables the jump^37^, resulting in a vertical acceleration that propels the insect as high as 49.3 ± 3.8 mm above the water surface (Fig. 3C–E, Supplementary movie S4). This is more than 15 times its body length, compared with only 3.7 times reported for the freshwater *Limnoporus dissortis*^38^. The maximum acceleration of 389 ms^−2^ observed for *Halobates* is also significantly higher than that of the freshwater *Aquarius paludum* (78 ms^−2^)^36,37^. The ability of *Halobates* to reach such remarkable acceleration can be attributed to the increased leg length relative to its body size and the evolution of their leg plan, driven by a key patterning gene^39,40^, where the middle legs are much longer than the hind legs, allowing it to generate rapid movements.

We explain the propulsion hydrodynamics of *Halobates* on the basis of Laplace pressure (*P*), generated due to the curvature of the air-water interface beneath the legs of the insect, given by $P=\gamma \times (1/R)\cos \,{\theta }_{{\rm{o}}}$, where *γ* is the surface tension of seawater, *R* is the axis-symmetric radius of curvature of the air-water interface, and ${\theta }_{{\rm{o}}}$ is the intrinsic contact angle at the solid-liquid-vapor interface. We refer the reader to Supplementary Fig. S3 to see the force body diagram at the interface of insect leg and the seawater. The surface tension of seawater depends on salinity, temperature, and organic contamination; here, we use the average value, $\gamma \,$= 70 mN/m. Based on *P*, we estimated the maximum upward capillary force (also known as the breakthrough pressure) that *Halobates* could generate by substituting for *R*, the radius of the middle legs, *R* = *r* and *θ* = 0° as $F=P\times (2r\times L)$, where *r* is the average radius of the legs and *L* is the total length of the legs in contact with water. The maximum capillary force was estimated to be $\,F\le 2\gamma L$ = 2.24 ± 0.14 mN, which theoretically could propel an insect weighing *m* = 4.7 ± 0.5 mg at accelerations of around 468 ms^−2^. Indeed, our high-speed experimental observations indicate that *Halobates* accelerates at 389 ms^−2^ off the water surface, which was within 25% of our prediction. We note that the insect might not exhibit its peak performance during our laboratory experiments and that performing such experiments in the natural setting of the open ocean is beyond the scope of this work.

Following a controlled jump, *Halobates* typically lands at a speed of 1 ms^−1^, a velocity similar to that observed during the jump, usually with its hind legs touching the surface of the water first. Remarkably, the insect does not penetrate the water surface upon landing despite the high vertical velocity at which it falls (Supplementary movies S4, S5), which is about 300 body lengths per second. As the insect lands, its two hind legs bend slightly to keep it afloat. Next, as the insect’s body impacts the water, the velocity diminishes significantly. Most of the energy is therefore dissipated by the insect’s body and not its legs, which suggests a possible air cushioning effect^41^, likely aided by retention of air by belly hairs (Fig. 1G,H). Locally, the impact configuration is similar to the bottom of a liquid drop impacting on a solid surface^42^. The air lubrication balances the inertia of the drop (here the insect body) with the viscous stress in the flow out of the thin air-layer separating the two surfaces. The thickness of this air layer δ is characterized with a Stokes number *St* = *µ*_g_ /(*ρ*_body_ ${l}^{{}^{{\prime} }}$ *U*), as $\left(\frac{\delta }{{l}^{{\prime} }}\right)=4.3\,S{t}^{2/3}$^43,44^. For *Halobates*, ${l}^{{}^{{\prime} }}$ = *w*/2 ≈ 2.3/2 mm and *U* ≈ 1 m/s, we get *δ* ≈ 6 µm. In our case the length and width of the body are different and the smaller of the two dimensions is the appropriate one to use for the escaping air. The air cushioning effect is hence more pronounced in sea striders with a lower body aspect ratio (length/width) than in cigar-shaped freshwater striders with a higher body aspect ratio (Table 1). This effect helps prevent wetting between the hairs and acts independently of the surface tension on the hairs penetrating the free surface^45^. For the superhydrophobicity to break down and water to penetrate inside the water-repellent plastron, the impact velocity of the insect would have to be higher than the threshold of 8 ms^−1^^45^, a value much higher than the typical landing velocity of *Halobates*. Furthermore, the microtrichia on the belly comprise hairs that are typically 1.5–2 µm in length, with ~1 µm spacing and stem diameter ~260 nm, affording *Halobates* a robust plastron (Fig. 1G,H and also see ref. ^18^), which also resists the drainage of air on impact, thereby enhancing the cushioning effect. This is known from the studies of drop impacts onto arrays of pillars over a range of spacings, including those similar to the plastron of *Halobates*^46^. To assess whether the hairs (or setae) might bend during the cushioning effect, we compared the viscous stress generated by the air-flow during the impact against the elastic stress required to bend the hairs by estimating the dimensionless Cauchy number, ${C}_{\gamma }$, given by the formula ${C}_{\gamma }={\mu }_{air}{U}_{air}{Z}^{3}/(2EI)$. Here, ${\mu }_{air}$ = 1.8 $\times$ 10^−5^ a.s is the dynamic viscosity of air, ${U}_{air}$ < 1 ms^−1^ is the radial air-speed out of the narrow gap estimated based on our recent report^47^, *Z* $\approx$ 1.5 µm is the representative length of microtrichia, *E* $\,\approx \,$9 GPa is the characteristic Young’s modulus for hairs from the work of Seale *et al*.^48^, and $I=\pi {d}^{4}/64$ is the second moment of the cross-sectional area of the hair assuming it to be a cylinder of diameter, *d* $\approx \,260$ nm. We find that ${C}_{\gamma }\ll 1$ for the *Halobates* hair, which demonstrates that the radial air-speed out of the narrow gap is insufficient to bend them.

**Table 1:** Habitat and body dimensions of water striders.

Species	Habitat	Body length (*l*) range (mm)	Body width (*w*) (mm)	Body mass (mg)	Estimated body aspect ratio (*l*/*w*)	References
*Halobates germanus*	open ocean	3.4 (f)–4.0 (m)	1.8 (m)–1.9 (f)	4–5	2.0	Present study and^72^
*Halobates micans*	open ocean	3.8 (f)–4.5 (m)	2.1(m)–2.3 (f)	unreported	1.9	^72^
*Halobates sericeus*	open ocean	3.0 (f)–3.6 (m)	1.5 (m)–1.7 (f)	unreported	2.0	^10,72^
*Halobates sobrinus*	open ocean	3.9 (f)–4.4 (m)	2.0 (m)–2.4 (f)	unreported	1.9	^10,72^
*Halobates splendens*	open ocean	4.3 (f)–5.0 (m)	2.3 (m)–2.4 (f)	unreported	2.0	^10,72^
*Halobates hayanus*	coastal	4.0 (m)–4.4 (f)	1.8 (m)–2.5 (f)	4–5	1.9	Present study and^73^
*Aquarius paludum*	freshwater	14–16	2.8–3.1	30.0–32.0 (m) 50.0–50.6 (f)	5.0	^36^
*Gerris buenoi*	freshwater	6.6 (m)–8.1 (f)	1.3–1.6	12.2	5.0	^74^
*Aquarius remigis*	freshwater	12–16	3–4	49.3 ± 2.7	4.0	^27,75^
*Limnogonus fossarum*	freshwater	11	3	12.36 ± 1.99	3.6	^76^

Note: Dimensions are either taken directly or estimated based on data presented in the references. Female (f) and male (m). *Gerris*, *Aquarius* and *Limnogonus* are all members of the subfamily Gerrinae (Family Gerridae).

### Implications of reduction in body mass and size

From our experimental studies we found that the impressive dynamic capacity of *Halobates* can be attributed to its low body mass ($\approx$5 mg), size, and body aspect ratio as compared with members of the Gerrinae which comprise the most primitive species of the Gerridae (Table 1). We estimated that *Halobates* could exert a maximum force of 2 mN during a jump (Fig. 3), in contrast to the 3mN force exerted by a much larger (40 mg) freshwater water strider species (Table 1)^27,28^. Thus, the force exerted on the water by the jump of *Halobates* is 33% weaker than the force exerted by the fresh water strider. In this context, the work of Alexander^49^ on the maximum force exerted by animals, including jumping forces for insects down to <1 mg in mass is relevant. When scaled to body mass, he found that acceleration (force-exerted/body-mass, m.s^−2^) is comprised within the allometric power laws defined by 20 *m*^−1/3^ and 0.5 *m*^−1/3^, where *m* is mass (kg)^49^. The acceleration expected from these allometric boundaries for an insect of the mass of *Halobates* ($\approx$5 mg) should range from 29–1169 m.s^−2^, substantiating our laboratory observation of a value of 398 m.s^−2^ for *Halobates* (Fig. 3E). *Halobates* typically took 7 ms to achieve its maximum velocity during a jump, in contrast to 20 ms required by the freshwater species, a reaction that is almost three-times faster. The maximum estimated kinetic energy of *Halobates* while moving at its maximum speed is 2.4 µJ, nearly 13 times less than that required by the freshwater *Aquarius paludum* (31 µJ)^36,37^. We therefore conclude *Halobates* to exhibit significantly advanced dynamics that not only improve their energy conservation but also provide efficient and effective escape maneuvers for avoiding predators and breaking waves that may push them underwater.

To further underscore the importance of the reduction in body mass and size, we estimated ratio of the weight of *Halobates* to the force due to the surface tension, defined as the Baudoin number (*Ba*), which is an indicator of the safety margin for the insect^50^ to remain at the water surface:

$$Ba=\frac{mg}{\gamma L},$$

where *m* is the mass of *Halobates*, *g* is the gravitational acceleration, $\gamma \,$is the surface tension of seawater and *L* is the total length of the legs in contact with seawater. In its typical resting state, *Halobates* places its middle and hind legs on the water surface, resulting in *L* ≈ 16 mm. Given that the average weight, *m*, of *H. germanus* is 4.7 ± 0.55 mg, *g* at the sea level is 9.81 m.s^−2^ and $\gamma \,$= 70 mN.m^−1^^51^, we estimate *Ba* = $4\times {10}^{-2}$. This low magnitude of *Ba* suggests that the insect can bear nearly 25 times its own body weight without sinking its legs, even though its body is denser than water. In a static position, *Halobates* can therefore efficiently support its weight by exploiting the surface tension of water. The absence of wings in *Halobates* species, unlike in many of its limnic relatives, is also a specific adaptation that further reduces its body weight. A strong correlation between the size of the insect’s body and the margin of safety has also been observed in other water-walking insects, but the order of magnitude reduction of the body mass of *Halobates* (Table 1) significantly increases the safety margin imparted by the interfacial forces under static conditions by an order of magnitude compared with those of many other surface dwelling freshwater heteropteran species of *Mesovelia*, *Hydrometera*, *Microvelia* and *Gerris*^30,52^.

### Predator avoidance

When exposed to a simulated predatory threat, *Halobates* demonstrated an impressive short reaction time of ~12 ms between the predatory signal and initiation of a jump (Fig. 3, Supplementary movie S5)^36,38^. This response time is almost twice faster than the average take-off time of 25.4 ± 12.8 ms (average reaction time to leave the water surface: 124.8 ± 18.6 ms) reported for a winged freshwater species *Limnoporus dissortis* following a predatory threat^38^. The escape strategy employed by *Halobates* seems to rely primarily on an instantaneous jump reflex coupled with a high-velocity vertical jump. In addition to this, *Halobates* can execute various other jump trajectories with variable speeds, distances and directions, indicating the versatility of their response to predators. This versatility has been shown to be particularly important in avoiding predation when *Halobates* gather in flotillas. The rapid propagation of responses from individuals at the edges of the flotilla generates an effective and flexible avoidance behavior that allows them to escape predation^53^. The ability to evade predators is crucial for the evolutionary success of *Halobates* in the open ocean where selective pressure exerted by predation is amplified several fold due to the lack of available shelters or places to hide.

### Surviving turbulence and accidental submergence

Turbulence was thought to be one of the major factors contributing to the absence of insects in the open ocean^54^. Although whitecaps cover only 3–4% of the ocean at any given time, these breaking waves continually disrupt the micro-layer of the ocean surface^55^. The evolutionary reduction of body size of *Halobates* to just 4–5 mm, comparable to the Kolmogorov or viscous length (about 5 mm)^56^, allows the insect to avoid turbulence from breaking waves. The energy density in the ocean dissipates rapidly towards scales approaching the Kolmogorov length such that the maximum size of turbulent eddies is of the order of 5 cm and contains only 1% of the maximum energy^56^. Hence, based on their small size, *Halobates* perceives an environment largely free of the potentially destructive turbulent velocity shear associated with the inertial range of scales in the Kolmogorov spectrum even during storms. A three-times larger freshwater species of *Aquarius*, *Gerris*. or *Limnogonus*. (Table 1) would be expected to experience much stronger velocity shear. Besides mastering survival in a turbulent ocean (Supplementary movie S6), *Halobates* also benefits from oceanic diffusion for dispersal^57^ to maintain the widest bio-geographical range.

**Figure 4:**
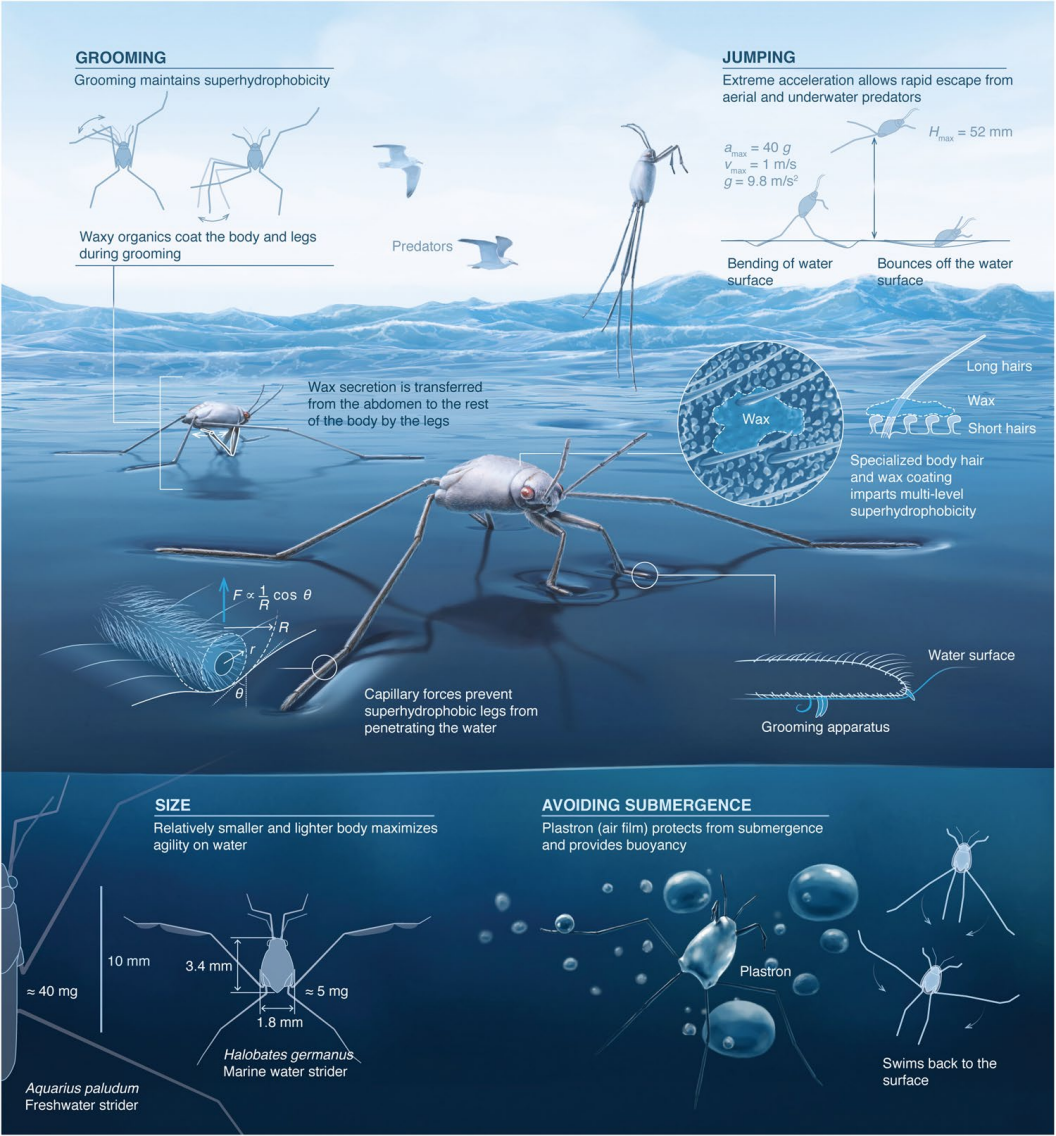
Summary of key adaptations of *Halobates* for oceanic life. *Halobates germanus* is one of only five insect species that managed to live on the perilous open ocean. Colonization of this habitat requires sophisticated and specific adaptations for a more efficient structural and functional body design that translates into behaviors and dynamics necessary to cope with various physical and ecological environmental threats. Oceanic *Halobates* evolved to have smaller and lighter water-repellent bodies, sets of specialized legs that can exploit the surface tension to support their weight and facilitate extraordinary environmental awareness, which are essential for versatile responses that allow them to survive in an environment characterized by turbulence, submersion and predation. (This figure was created by Xavier Pita, Senior Scientific Illustrator, KAUST).

During accidental submersion, the plastron plays a crucial role because the air layer held by the microtrichia allows *Halobates* to breathe underwater and to resurface rapidly due to increased buoyancy (Fig. 4, Supplementary Movie S7)^18,21,22^. While it was not possible to experimentally determine the plastron size, we applied scaling arguments to assess how it might vary with the dimensions of the insect body. If we assume the insect’s body (without legs) to be represented by a cylinder of radius, *r*, and length, *l*, the surface area is $S=2\pi rl$ and the mass of the insect, *m*, is related to its volume, *V*, by its density, $\rho$, as, $m=\rho \pi {r}^{2}l$. Let’s assume the thickness of the plastron to be $\Delta z$, such that the volume of the plastron is ${V}_{{\rm{P}}}=S\times \Delta z=2\pi rl\Delta z$. Thus, the ratio of the plastron to the mass of the insect becomes, ${V}_{{\rm{P}}}/m=2\pi rl\Delta z/\rho \pi {r}^{2}l$, which scales with the width of the insect as $\propto {r}^{-1}$. The small body size of *Halobates* therefore results in a larger plastron relative to body weight, thereby increasing its buoyancy (Table 1). In addition, the air trapped within the plastron also reduces the drag on the body of the insect as it rises through the water^58^. Once the insect resurfaces, it dries quickly as droplets of water roll off its superhydrophobic hairs. However, prolonged submersion could impact the water-repellent characteristics due to structural rearrangement and contamination^59^, making it difficult for the insect to regain the surface. If it resurfaces and has the opportunity to groom and dry itself, the superhydrophobic properties of the hairs can be restored (Supplementary movies S2 and S3).

Without wings or the ability to dive, *Halobates* individuals are dispersed across the ocean via surface currents and winds acting at the sea-air interface. From the evolutionary perspective, the water-evading mechanisms developed by *Halobates* are significant not only for survival in a turbulent and dynamic ocean, but also for transport and dispersal of individuals to new locations.

## Conclusion

We investigated an open-ocean *Halobates germanus* and a coastal *H*. *hayanus* to explore their morphological, behavioral and bio-mechanical characteristics that underpin their oceanic lifestyle and successful occupation of the ocean. We found that together with the specialized body hair covering, highly specialized behavioral adaptations are crucial to enable them to overcome major environmental and predatory hurdles prevalent in the open ocean. The key specializations that enabled five species of *Halobates* to be the only insects to colonize and proliferate across the open ocean (Fig. 4) could be facilitated by two major adaptations. The first is the insect’s reduced body size and the second is the combination of nested anatomical, chemical and behavioral features that confer robust superhydrophobicity, which essentially protects them from wetting or drowning.

Body size is a key morphological trait with crucial implications for many aspects of an animal’s biology, behavior, ecology and ultimately its evolutionary success^60^. The chief advantage of oceanic *Halobates* over many of its more primitive freshwater relatives is the much-reduced body size with a significantly reduced body aspect ratio (Table 1). This size reduction could have three possible consequences with crucial adaptive values to succeed in the open ocean, namely (i) reduced visibility to predators, (ii) increased stability and geometry of the plastron for trapping air, allowing them to breathe under water and gain buoyancy for re-surfacing, (iii) enhanced jumping capacity to evade predators by reducing reaction times and increasing acceleration. Although many of the biological peculiarities of oceanic *Halobates* could not be attributed to one single feature, it is interesting to note that, unlike many terrestrial organisms that may require major genomic re-engineering to colonize the sea^61^, genomic adaptations in *Halobates* are limited to selection of only a few genes leading to extended leg lengths, which allow all Gerridae to walk on water^62^.

While the results presented here demonstrate the importance of superhydrophobicity, grooming, and reduction in body size and mass to explain the success of oceanic *Halobates* in colonizing the ocean, they do not explain why coastal *Halobates* species with similar characteristics could not survive in the open ocean. We submit that while adaptations we report are essential conditions for the success of oceanic *Halobates*, their success also requires other important life-history adaptations previously reported, such as winglessness, ability to lay eggs on floating substrates, longer oviposition periods, slower growth rates and longer life spans compared with coastal and related freshwater species^14–16^. This extended life-history strategy has been suggested to offer advantages in finding food and mates, leading to reproduction even after wide dispersal due to turbulent environmental events^57^. Yet, an extended life-history strategy can only allow insects to colonize the open ocean if they can survive in that environment, and such survival requires superhydrophobicity, grooming, and reduction in body size and mass.

While our investigation sheds light on certain significant aspects of *Halobates*, several questions remain unanswered. For instance, it remains unclear why other insects that exhibit superhydrophobicity and small mass did not colonize the open ocean. Perhaps, some of those questions might be properly addressed through field-studies in the open oceans, but the complexity of performing similar experiments under those circumstances poses significant challenges that render this goal unfeasible with existing technology. Our study, however, is a step towards better understanding adaptations that are likely products of evolutionary selection of traits resulting in a fine-tuned ocean-worthy prototype, and can help to explain the unique evolutionary feat of the colonization of the open ocean by just five species of *Halobates*.

We also hope that our investigation on this widespread and unique insect will inspire rational designs of materials and processes across a wide spectrum of applications. Mushroom-shaped microtrichia of *Halobates* could inspire coating-free liquid repellence and the prolonged entrapment of air in micro-textures could unleash the potential of common materials in drag reduction^63–66^, mitigating cavitation^67^, and desalination^68,69^. Grooming strategies of *Halobates* might inspire new designs for preventing membrane fouling that commonly affect the efficiency of desalination processes^70,71^. The cushioning ability of *Halobates* towards minimizing the damage/bounce when impacting the water surface could inspire damage-proof designs for landing of micro-robotic vehicles. With recent advances in platforms for high-resolution imaging and tomography, micro and nano fabrication, and additive or 3D manufacturing, the translation of these insights into new technologies might be possible in the near future.

## Methods

### Animal collection and acclimation

Individuals of the species *Halobates germanus* White, 1883 were collected from an offshore sampling station in the Central Red Sea (22°20′32.6″N 39°05′23.6″E) during day time using a neuston net towed at 1–2 knots. *H. hayanus* White, 1883 individuals were collected from a mangrove ecosystem near the shore of the Central Red Sea (22°20′32.6″N 39°05′23.6″E)^77^. The number of individuals caught offshore was always low (2–8 individuals per tow), thereby restricting their inclusion to selected experiments. After collection, insects were transferred to a bucket filled with seawater and immediately transported to the laboratory. They were then gently moved to a glass aquarium filled with seawater collected from the sampling site. Water and air temperatures were maintained at 25 °C, and gentle illumination was provided by a lamp with a 12D:12 N cycle. The insects were always acclimated to laboratory conditions for at least 12 hours prior to high-speed imaging. During the acclimation period, they were fed with brine shrimp *Artemia salina*. While collected insects consisted of a mixed population of adult males, females and nymphs, we selected only adults for the behavioral studies. For microscopic imaging and wax extraction studies, insects were immediately processed by following respective protocols.

### Body surface and hair microstructure examination

Body surfaces and hair structures of *Halobates* were examined with scanning electron microscopy (SEM) and environmental SEM (ESEM). For SEM, samples were fixed and prepared following the standard protocol for biological material. After fixation, samples were dehydrated in an increasing series of ethanol and subjected to critical point drying. They were then mounted on aluminum stubs and coated with a 4-nm Au layer before observation by SEM (FEI Quanta 600).

### Wax extraction and analysis

The collected insects were rinsed with DI water for about 2 minutes and then dried with nitrogen gas and air at ambient temperature (20 °C) for 20 minutes. They were then kept at 4 °C for 20 minutes and in pure nitrogen gas for 10 minutes at 20 °C. The resulting sample was weighed (1.224 g) and then extracted in 10 mL of toluene for 10 minutes. The toluene extract was filtered and 8 mL of the extract was collected in a separate vial. The toluene was then allowed to evaporate at 80 °C for 12 hours. Next, 1 mL of fresh toluene was added to the extract, and an aliquot (5 μL) was analyzed by gas chromatography – mass spectroscopy (GCMS) (Agilent 7890A-5975C). The remainder of the sample was again completely evaporated at 100 °C for 3 hours and then analyzed by GCMS.

### Measurement of contact angles to assess wettability

We characterized superhydrophobicity of *Halobates* by measuring advancing, receding, and advancing static contact angles of deionized water on various parts of their body using a Kruss Drop Shape Analyzer-DSA100. Typical experiments involved applying 2-μL droplets of deionized water at a rate of 0.2 μLs^−1^ from a metallic capillary dispenser while a camera recorded the images. The images were analyzed using the Advance and ImageJ software (National Institutes of Health, USA), an open source program^78^ that required fitting precise spheres into the drop and/or tangents at the solid-liquid-vapor contact line to deduce contact angles. Each data point presented here is an average of at least five measurements.

### Static position stability and deformation at the interface

To visualize deformation of the water surface by *Halobates*, we used a projection of color bands^79^. We illuminated the surface of the water using a home theater projector (Epson EX9200 Pro) and a custom strip pattern. The projector was focused towards the camera at an oblique angle to reproduce the stripped pattern on the water surface. This specific setup showed the water surface as a dark plane with intense reflection. The position and movement of the insect were recorded with a color high-speed camera (Photron Fastcam SA3), typically at 1000 frames per second (fps). The insects were illuminated by a backlight diffuser from the back of the camera using a Sumita metal halide cool light source (Sumita Optical glass, Inc.). This resulted in the insects being seen as black shadows projected on the camera.

### Visualization of behavioral responses

We tested responses of *Halobates* to a variety of challenges they might experience in the marine environment using simulated experimental setups. We chose the most common and precarious situations that would pose threats to the insect’s survival, namely, predators and rain storms or breaking waves that disrupt the physical properties at the sea surface (air-sea interface). For each experiment, a single or up to nine adult *Halobates* adults were placed in an aquarium for observation. Responses of the insects were recorded using a Photron Fastcam SA3 high-speed camera that captures images at speeds between 50 to 1000 fps. Different lenses with focal lengths ranging from 50 to 100 mm were used according to the position of the insect to achieve the desired field of view and magnification. Illumination was provided by either a video projector (Epson EX9200 Pro) or a Sumita light source (Sumita Optical Glass, Inc.).

Predatory threat signals from either below or above the sea surface were created by hitting the outer wall of the aquarium at different positions to generate a mechanical disturbance. To simulate fine rain or splashing waves, we produced micron-sized droplets (typical size of 30 µm) at rates ranging from 10 to 6000 drops per second using a Microdrop nozzle (MD-K-130) and a dispensing unit (MD-E-3000). To simulate breaking waves, we poured suitable volumes of water directly on top of the insects. The flow rate was typically 50 mL per second, which is comparable to breaking waves on a rough ocean.

### Estimation of behavioral and biomechanical/kinetic parameters from video footage

To acquire quantitative data of different parameters we analyzed each video sequence frame-by-frame using the ImageJ software to identify positions of the insect in response to different threats. We then converted the number of frames an individual spent performing a particular behavior to milliseconds. The pixel-to-μm conversion factor was calculated by measuring objects of known size in the picture.

## Supplementary information

Supplementary Information.

Movie S1.

Movie S2.

Movie S3.

Movie S4.

Movie S5.

Movie S6.

Movie S7.

## Data Availability

All data needed to evaluate the conclusions in the paper are present in the paper and/or Supplementary Materials. Additional data related to this paper may be requested from the authors.
