# Supplementary material for: Superhydrophobicity and size reduction enabled Halobates (Insecta: Heteroptera, Gerridae) to colonize the open ocean
Source: Sci Rep. 2020 May 8;10:7785. doi: 10.1038/s41598-020-64563-7 (PMC7210887; doi:10.1038/s41598-020-64563-7)
Supplement: Supplementary file 1 — Supplementary Information. [file 41598_2020_64563_MOESM1_ESM.docx]

**Supplementary Information**

Superhydrophobicity and size reduction enabled *Halobates* (Insecta: Heteroptera, Gerridae) to colonize the open ocean

G. A. Mahadik^1^, J. F. Hernández-Sánchez^2^, S. Arunachalam^3^, A. Gallo Jr.^3^, L. Cheng^4^, A. S. Farinha^3^, S. T. Thoroddsen^2^, H. Mishra^3*^, Carlos M. Duarte^1^

^1^King Abdullah University of Science and Technology (KAUST), Biological and Environmental Science and Engineering (BESE) Division, Red Sea Research Center (RSRC), Thuwal, 23955-6900, Saudi Arabia.

^2^King Abdullah University of Science and Technology (KAUST), Physical Science and Engineering (PSE), Thuwal 23955-6900, Saudi Arabia.

^3^King Abdullah University of Science and Technology (KAUST), Biological and Environmental Science and Engineering (BESE) Division, Water Desalination and Reuse Center (WDRC), Thuwal 23955-6900, Saudi Arabia.

^4^Scripps Institute of Oceanography, University of California San Diego, La Jolla,

CA 92093-0202, USA

*[Himanshu.Mishra@kaust.edu.sa](mailto:Himanshu.Mishra@kaust.edu.sa)

**Section S1. Hairs of Gerridae**

The fine structure of body hair layer in six families of semi-aquatic bugs, including the Gerridae to which *Halobates* belongs, has been reviewed by Andersen^1^ with detailed descriptions of hair layer morphology. In the freshwater *Gerris lacustris*, the two types of hairs measure 40-60 and 80-100 μm-long, with densities of 3,000-5,000 and 100-300 mm^-2^, respectively.  In the two *Halobates* species studied (a coastal *H. flaviventris* and an oceanic *H. micans*), the two types of hairs are much shorter with the common type measuring 20-30 μm with densities of 8,000-12,000 mm^-2^. The length was not reported for the second longer hair type with a density of 4,000-5,000 mm^-2^. The densities of macro-hairs in *Halobates* are much higher for both hair types. The micro-hair layer is distributed all over the body in most species studied but differs greatly between species in size, shape and orientation. However, the structure of the micro-hair in *Halobates* is completely different from all other species studied in being club-shaped instead of filiform or simple peg-shaped, first discovered by Cheng^2^. The micro-hairs on the dorso-lateral surface of *Gerris lacustris* are 2-6 μm long with densities reaching 5-8$\times$100,000 mm^-2^ on the metasternum (density was not reported for the dorso-lateral surface). In *Halobates* the micro-hairs are much shorter and measure 1.5-3.0 μm with densities reaching 6-7$\times$100,000 mm^-2^. This is much lower than that of 2.2-2.4$\times$1,000,000 mm^-2^ calculated by Hinton^3^ based on micrographs in Cheng^2^.

**Section S2. The Cassie-Baxter Model**

In the resting position, only a fraction of the surface area of the insect’s leg is in contact with the water and the rest of the leg is surrounded by air. We estimated the solid-liquid and solid-air surface areas by measuring the (advancing) apparent contact angles of water, *θ*_r_ , as shown below (Fig. S1).


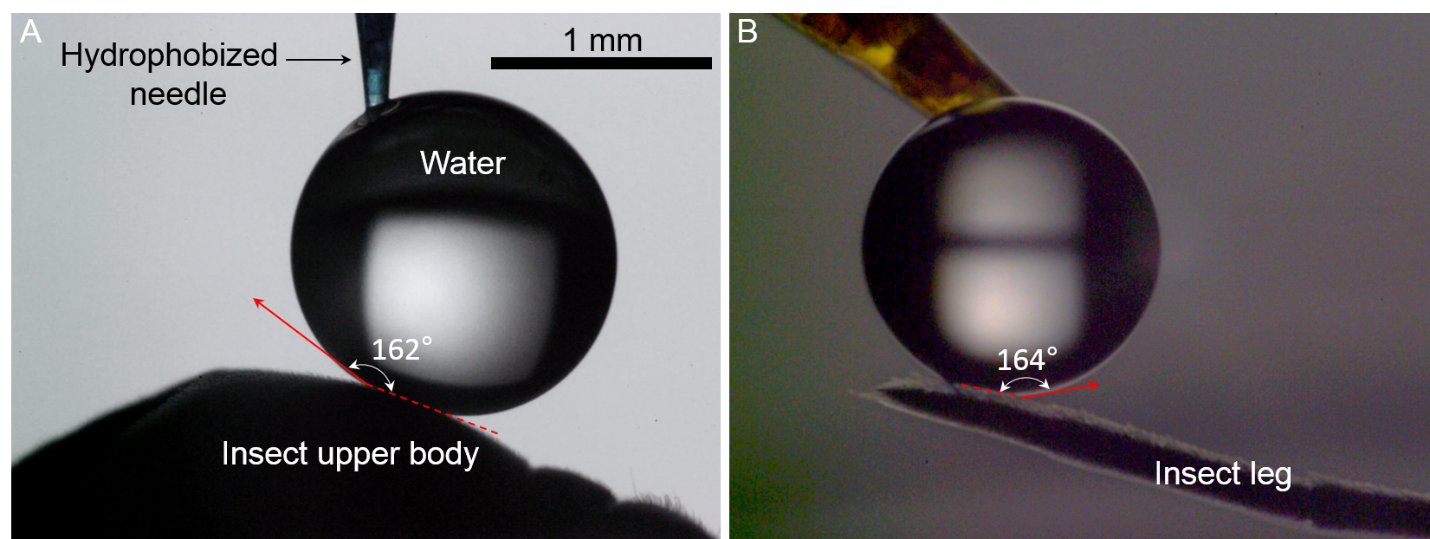


**Fig. S1.** Behaviors of water droplets on the body and leg of *Halobates*.

Since the sizes of the hierarchical structures, including the hairs and setae of *Halobates,* were much smaller than the size of the droplets of water used to characterize wetting, we could employ the Cassie-Baxter model ^4^ to deduce the solid-liquid and solid-air surface area fractions:

$\cos\theta_{r}={}_{\mathrm{LS}}\cos\theta_{o}-{}_{\mathrm{LV}}$, (1)

where *θ*_r_ is the apparent contact angle, *θ*_o_ is the intrinsic contact angle of the liquid on a smooth and flat wax surface (*θ*_o_ ≈105°)^5^, and *φ*_LS_ = *A*_LS_/*A*_H_ and *φ*_LV_ = *A*_LV_/*A*_H_ are the ratios of real liquid-solid (*A*_LS_) and liquid-vapor (*A*_LV_) areas compared to the projected horizontal area (*A*_H_) ^4,6^.


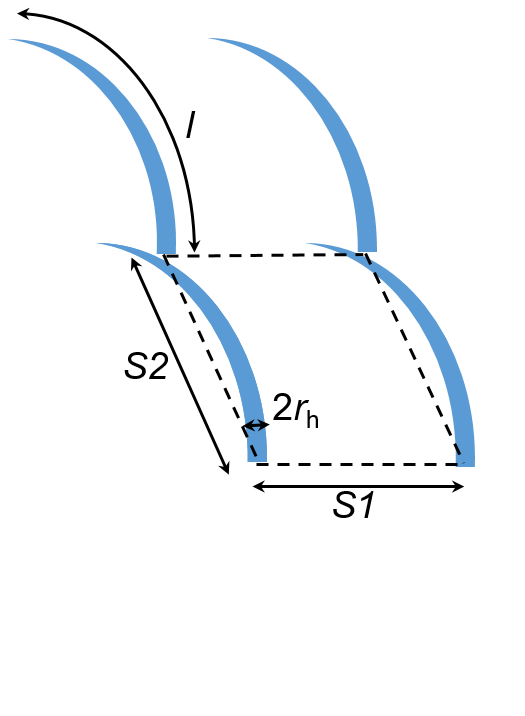


**Fig. S2.** A schematic representation of the distribution of hair on the leg of *Halobates*.

Based on the apparent contact angle on the leg (*θ*_r_ ≈164°) and hair distribution (Fig. S2), we calculated *φ*_LV_ and *φ*_LS_ as

$\phi_{\mathrm{LV}}=\frac{(S1\times S2)-\pi r_{h}l}{S1\times S2}\approx95\%$ (2)

and

$\phi_{\mathrm{LS}}=\frac{\pi r_{h}l}{S1\times S2}\approx5\%$ , (3)

where *S1* and *S2* are the pitches along the two axes, *l* is the length of the hairs in contact with water, and *r*_h_ is the radius. Using scanning electron microscopy, we estimated *S1* ≈ 18.2 μm, *S1* ≈ 8.5 μm, and *r_h_* = 2.2 μm (Fig. 1).

We also calculated the capillary length, $\lambda_{c}$, defined as the ratio of the force due to surface tension and gravitation as ^7^

$\lambda_{c}=\sqrt{\gamma/{\rho g}}$ , (4)

where *γ* is the surface tension, *ρ* is the density and *g* is the acceleration due to gravity. For water, the capillary was estimated to be $\lambda_{c}=2.7 \mathrm{mm}$ at 293 K and 1 atm, and we used droplets smaller than this dimension in our wetting experiments to understand the capillary phenomena, ignoring the inertial effects.

**
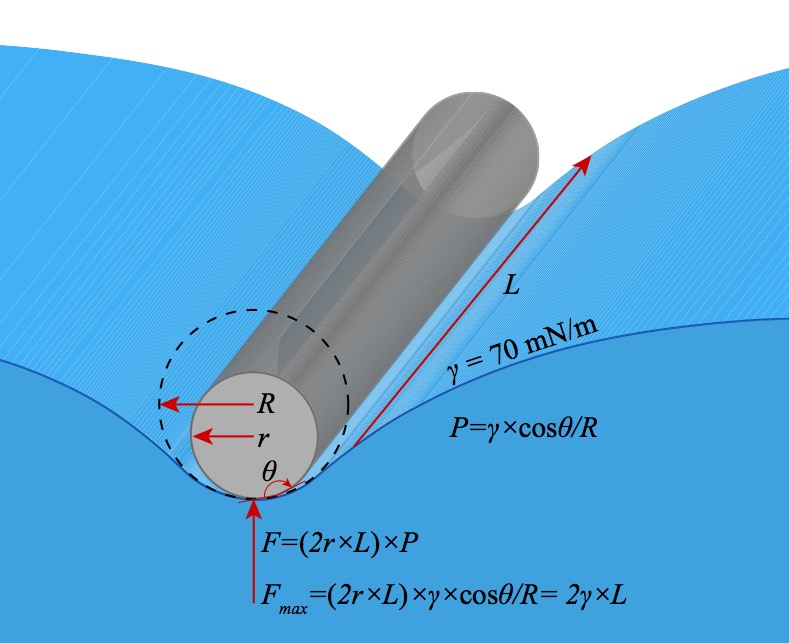
**

**Fig. S3**. This schematic represents a superhydrophobic leg of a water strider pressing against the surface of seawater. As a result, the air-water meniscus gets curved generating Laplace pressure, *P*, which for this (cylindrical) geometry depends on the curvature of the meniscus, *R*, the surface tension, $\gamma$, and the angle at the solid-liquid-vapor interface, $\theta$, as $P=\gamma\left( 1/R \right)\cos\theta$. The upward force due to this pressure is calculated by multiplying the pressure with the projected area of the cylindrical leg, which is $2r\times L$, where *r* is the radius and *L* is the total length of the insects’ legs. The maximum force is achieved when $R\to r$ and $\theta\to0^{\circ}$, and it is given by $F_{max}=P\times\left( 2r\times L \right)=2\gamma L$. (Please note: we have not shown hairs on the leg)

**Table S1**. Composition of organic compounds extracted from *Halobates hayanus*

| **Relative Conc. (%)** | **Compound** | **Mol. Weight (amu)** | **RT (min)** |
| --- | --- | --- | --- |
| 18.57 | 1-heptacosanol/ 1-tetracosanol/ | 396.4/354.4 | 34.2 |
| 15.68 | 1,21-Docosadiene | 306.3 | 32.7 |
| 12.08 | 3-Ethyltetracosane/ 11-Butyldocosane/ 5,14-Dibutyloctadecane | 366.4 | 36.4 |
| 10.51 | 1-Docosanol/ 1-tetracosanol | 354.4/354.4 | 27.4 |
| 6.17 | cis-5,8,11,14,17-Eicosapentaenoic acid methyl ester | 316.2 | 32.0 |
| 5.28 | 9-methyl-nonadecane | 282.3 | 28.7 |
| 4.83 | Oleyl Alcohol | 268.3 | 33.5 |
| 4.72 | 9-Nonadecene | 266.2 | 33.6 |
| 3.06 | Methyl -4,7-octadecadienoate | 294.2 | 31.6 |
| 2.64 | 3-ethyl-tetracosane | 366.4 | 36.6 |
| 1.61 | Heptacosane | 380.4 | 28.8 |
| 1.05 | Tetracosane | 338.4 | 29.1 |
| 1.02 | Hexacosane | 366.4 | 29.8 |
| 0.81 | 1-Hexacosene | 364.4 | 30.1 |
| 0.78 | 1,21-Docosadiene | 306.3 | 42.6 |
| 0.74 | Acetate (Z)-11-Hexadecenol | 282.2 | 27.0 |
| 0.69 | Oleyl Alcohol | 268.2 | 26.5 |
| 0.68 | 9-octyl-Heptadecane | 352.4 | 38.4 |
| 0.61 | 1-Nonadecene | 266.3 | 30.2 |
| 0.60 | Tricosane | 324.4 | 34.6 |
| 0.60 | 9-methyl-nonadecane, | 282.3 | 48.7 |
| 0.57 | 1-Octacosanol | 410.4 | 34.4 |
| 0.54 | heptadecyl-oxirane, | 282.3 | 22.6 |
| 0.52 | n-1-Tetracosanol | 354.4 | 27.1 |
| 0.38 | Docosane | 310.4 | 41.6 |
| 0.34 | Tetradecanal | 212.2 | 14.1 |
| 0.34 | Dimethyl 6-methoxyquinolinate | 225.1 | 47.2 |
| 0.32 | (E,E,E)-1,4,8-Dodecatriene | 162.1 | 26.1 |
| 0.27 | Octadecane | 254.3 | 27.7 |
| 0.26 | Methyl eicosa-5,8,11,14,17-pentaenoate | 316.2 | 40.3 |
| 0.23 | 26-Nor-5-cholesten-3.beta.-ol-25-one | 386.3 | 44.2 |
| 0.21 | 1-iodo-hexadecane | 352.2 | 29.5 |
| 0.19 | 2,6,10,14-tetramethyl-hexadecane | 282.3 | 29.6 |
| 0.19 | cis-Bicyclo[10.8.0]eicosane | 278.3 | 20.3 |
| 0.18 | 2,4-dimethyl-2,6-heptadienal | 138.1 | 15.8 |
| 0.17 | 5-methyl-octadecane | 268.3 | 36.9 |
| 0.16 | (Z)-9-Tricosene | 322.4 | 22.3 |
| 0.16 | Triacontyl acetate | 480.5 | 45.1 |
| 0.16 | 2-methyl-octadecane | 268.3 | 25.4 |
| 0.15 | Octacosane | 394.5 | 30.7 |
| 0.14 | 10-methyl-Eicosane, | 296.3 | 23.6 |
| 0.13 | 1-Nonadecene | 266.3 | 25.1 |
| 0.12 | Tetradecanal | 212.2 | 15.3 |
| 0.11 | Heneicosane | 296.3 | 19.2 |

**Captions for movies S1 to S7**

**Movie S1.** A water drop bounces off the dorsal body surface of *Halobates germanus*. The hierarchical structures of the composite hairs confer the high contact angle characteristic of a non-wetting (superhydrophobic) surface. The drop diameter is ~1.7 mm. The video was recorded at 3000 frames per second (fps) and is played at 5 fps.

**Movie S2.** Side view of *Halobates hayanus* performing grooming. Grooming is a part of the natural behavioral repertoire of the insect and is essential to maintain the water-repellent properties of its body and legs. This video was recorded at 50 fps and is played at 30 fps.

**Movie S3.** Grooming sequence of *Halobates germanus* following continuous exposure to a micro droplet spray. This video was recorded at 50 fps and is played at real time

**Movie S4.** Close-up side view of the body and leg movement of *Halobates germanus* at take-off during a typical jump. The jump initiates with the pairs of middle and hind legs pushing on the surface of the water to create dimples, without piercing the surface, thereby maximizing the take-off speed. The legs leave the water’s surface, the tips at the end, attaining a tripod position as it propels into a near vertical jump and becomes airborne. Here, we measured a maximum acceleration of ~300 ms^-2^ (31*g*) on the body of the insect. After accelerating, the insect achieves a maximum speed of approximately 1 ms^-1^. The video was recorded at 1000 fps and is played at 10 fps.

**Movie S5.** A typical high-velocity jump sequence displayed by *Halobates germanus*, observed as an escape response to a simulated predatory signal*.* The reaction time between the impact stimulus and initiation of the jump is ~12-13 ms. An average jump lasts for ~7 ms and can propel the insect up to 49.26±3.8 mm above the water surface. This video was recorded at 1000 fps and is played at 10 fps.

**Movie S6.** Efficient reflex of *Halobates germanus* to flooding with a stream of water. The flow rate is approximately 90 mLs^-1^. The insect perceived the stream of water 12.0 ± 2.2 ms before the impact of the stream with the water’s surface and escaped from the turbulence by sliding on the surface along the wave. While this maneuver was not effective in avoiding the splashing of water on its body, it was effective for staying afloat. This video was recorded at 1000 fps and is played at 10 fps.

**Movie S7**. Formation of a plastron following entrapment of *Halobates* below the surface of the water as the insect is submerged. The flow rate of the water is ~270 mLs^-1^. The plastron increased the buoyancy of the insect. Irrespective of the volume of water poured on top of the inset during several tests, the plastron allowed the insect to resurface. The plastron also seems to reduce the drag on the insect’s body as it rises through the water. This video was recorded at 1000 fps and is played at 30 fps.

**References:**

1 Andersen, N. M. Fine structure of the body hair layers and morphology of th spirales of semiaquatic bugs (Insects, Hemiptera, Gerromorpha) in relation to life on the water surface. *Videnskabelige meddelelser fra Dansk naturhistorisk forening i Kjøbenhavn* (1977).

2 Cheng, L. Marine and freshwater skaters: differences in surface fine structures. *Nature* **242**, 132 (1973).

3 Hinton, H. Plastron respiration in bugs and beetles. *Journal of Insect Physiology* **22**, 1529-1550 (1976).

4 Cassie, A. B. D. & Baxter, S. Wettability of porous surfaces. *Transactions of the Faraday Society* **40**, 0546-0550, doi:DOI 10.1039/tf9444000546 (1944).

5 Holdgate, M. The wetting of insect cuticles by water. *Journal of Experimental Biology* **32**, 591-617 (1955).

6 Kaufman, Y. *et al.* Simple-to-Apply Wetting Model to Predict Thermodynamically Stable and Metastable Contact Angles on Textured/Rough/Patterned Surfaces. *Journal of Physical Chemistry C* **121**, 5642-5656, doi:10.1021/acs.jocc.7b00003 (2017).

7 Butt, H. J. & Kappl, M. *Surface and Interfacial Forces*. (Wiley-VCH Verlag GmbH & Co, 2010).
